# Supplementary material for: Hot spot prediction in protein-protein interactions by an ensemble system
Source: BMC Syst Biol. 2018 Dec 31;12(Suppl 9):132. doi: 10.1186/s12918-018-0665-8 (PMC6311905; doi:10.1186/s12918-018-0665-8)
Supplement: Supplementary file 2 — All relevant MATLAB code. A simple Matlab implement of our predictor. (ZIP 19,742 kb) [file 12918_2018_665_MOESM2_ESM.zip › Additional file 2/Read ASA/ASA Property/SKEMPI/2PCCB.txt]

 NetSurfP - Protein Surface Accessibility and Secondary Structure Predictions

  

|  |  |  |
| --- | --- | --- |
|  |  | NetSurfP - Protein Surface Accessibility and Secondary Structure Predictions  Technical University of Denmark |

  


---

```
# For publication of results, please cite:
# A generic method for assignment of reliability scores applied to solvent accessibility predictions.
# Bent Petersen, Thomas Nordahl Petersen, Pernille Andersen, Morten Nielsen and Claus Lundegaard
# BMC Structural Biology 2009, 9:51 doi:10.1186/1472-6807-9-51
#
# Column 1: Class assignment - B for buried or E for Exposed - Threshold: 25% exposure, but not based on RSA
# Column 2: Amino acid
# Column 3: Sequence name
# Column 4: Amino acid number
# Column 5: Relative Surface Accessibility - RSA
# Column 6: Absolute Surface Accessibility
# Column 7: Z-fit score for RSA prediction
# Column 8: Probability for Alpha-Helix
# Column 9: Probability for Beta-strand
# Column 10: Probability for Coil
E T  2PCCB                  1    0.723 100.225  -0.742   0.016   0.005   0.979
E E  2PCCB                  2    0.514  89.778   0.040   0.058   0.017   0.925
B F  2PCCB                  3    0.209  41.866  -0.073   0.018   0.019   0.964
E K  2PCCB                  4    0.464  95.486  -0.026   0.058   0.017   0.925
E A  2PCCB                  5    0.700  77.195   0.432   0.115   0.016   0.868
E G  2PCCB                  6    0.381  29.961  -0.758   0.058   0.017   0.925
E S  2PCCB                  7    0.422  49.505   1.346   0.058   0.017   0.925
B A  2PCCB                  8    0.217  23.891   0.292   0.782   0.003   0.216
E K  2PCCB                  9    0.740 152.239   1.842   0.858   0.002   0.139
E K  2PCCB                 10    0.447  91.886   1.991   0.782   0.003   0.216
B G  2PCCB                 11    0.079   6.186  -0.770   0.858   0.002   0.139
E A  2PCCB                 12    0.354  38.967   1.825   0.970   0.001   0.030
E T  2PCCB                 13    0.585  81.139   2.055   0.970   0.001   0.030
B L  2PCCB                 14    0.124  22.686   1.376   0.970   0.001   0.030
B F  2PCCB                 15    0.065  13.025   0.132   0.970   0.001   0.030
E K  2PCCB                 16    0.526 108.219   1.814   0.923   0.002   0.076
E T  2PCCB                 17    0.555  76.937   1.211   0.782   0.003   0.216
E R  2PCCB                 18    0.454 103.943   1.189   0.406   0.004   0.590
B C  2PCCB                 19    0.061   8.522  -0.048   0.181   0.016   0.803
E L  2PCCB                 20    0.350  63.993   0.567   0.278   0.093   0.628
E Q  2PCCB                 21    0.345  61.671   0.113   0.216   0.235   0.548
B C  2PCCB                 22    0.090  12.594   0.460   0.135   0.317   0.548
B H  2PCCB                 23    0.126  22.883   0.343   0.064   0.216   0.721
E T  2PCCB                 24    0.409  56.714   0.088   0.056   0.142   0.802
B V  2PCCB                 25    0.201  30.940   0.391   0.056   0.142   0.802
E E  2PCCB                 26    0.452  78.895   0.478   0.018   0.088   0.893
E K  2PCCB                 27    0.634 130.434  -0.064   0.018   0.047   0.935
E G  2PCCB                 28    0.681  53.595  -1.004   0.018   0.019   0.964
E G  2PCCB                 29    0.384  30.229  -0.991   0.018   0.019   0.964
E P  2PCCB                 30    0.647  91.753  -0.102   0.018   0.019   0.964
E H  2PCCB                 31    0.497  90.332  -1.311   0.018   0.047   0.935
E K  2PCCB                 32    0.414  85.119  -0.559   0.019   0.141   0.840
B V  2PCCB                 33    0.222  34.152  -1.115   0.019   0.141   0.840
B G  2PCCB                 34    0.177  13.930  -1.240   0.018   0.047   0.935
B P  2PCCB                 35    0.134  19.071  -0.351   0.018   0.019   0.964
E N  2PCCB                 36    0.342  50.054   0.179   0.018   0.047   0.935
B L  2PCCB                 37    0.073  13.421  -0.302   0.113   0.087   0.800
E H  2PCCB                 38    0.327  59.499  -0.093   0.113   0.087   0.800
B G  2PCCB                 39    0.248  19.518  -0.914   0.118   0.150   0.732
B I  2PCCB                 40    0.075  13.893   0.109   0.216   0.235   0.548
B F  2PCCB                 41    0.195  39.076  -0.981   0.199   0.152   0.649
E G  2PCCB                 42    0.354  27.868  -1.008   0.113   0.087   0.800
B R  2PCCB                 43    0.202  46.258   0.439   0.113   0.087   0.800
E H  2PCCB                 44    0.509  92.514   0.069   0.118   0.150   0.732
E S  2PCCB                 45    0.373  43.716  -0.342   0.118   0.150   0.732
B G  2PCCB                 46    0.260  20.423  -1.628   0.125   0.227   0.648
E Q  2PCCB                 47    0.400  71.422  -1.031   0.118   0.150   0.732
B A  2PCCB                 48    0.275  30.360  -0.305   0.113   0.087   0.800
E E  2PCCB                 49    0.674 117.730  -1.005   0.053   0.043   0.903
E G  2PCCB                 50    0.524  41.199  -1.891   0.053   0.043   0.903
B Y  2PCCB                 51    0.143  30.474  -0.437   0.052   0.084   0.864
E S  2PCCB                 52    0.498  58.377  -0.230   0.113   0.087   0.800
B Y  2PCCB                 53    0.165  35.346  -0.516   0.184   0.043   0.773
B T  2PCCB                 54    0.178  24.675  -1.671   0.339   0.016   0.645
E D  2PCCB                 55    0.554  79.846  -0.051   0.717   0.014   0.269
B A  2PCCB                 56    0.248  27.352  -1.675   0.622   0.015   0.363
B N  2PCCB                 57    0.108  15.870  -0.791   0.622   0.015   0.363
E I  2PCCB                 58    0.451  83.527  -0.295   0.622   0.015   0.363
E K  2PCCB                 59    0.688 141.542  -0.381   0.502   0.002   0.495
E K  2PCCB                 60    0.398  81.807  -0.474   0.246   0.004   0.750
E N  2PCCB                 61    0.591  86.464  -1.454   0.058   0.017   0.925
B V  2PCCB                 62    0.203  31.155   0.337   0.052   0.084   0.864
E L  2PCCB                 63    0.425  77.909   0.249   0.020   0.205   0.775
B W  2PCCB                 64    0.143  34.488   0.823   0.004   0.197   0.799
E D  2PCCB                 65    0.297  42.769   1.020   0.005   0.045   0.951
E E  2PCCB                 66    0.409  71.400   0.323   0.923   0.002   0.076
E N  2PCCB                 67    0.634  92.744   0.465   0.923   0.002   0.076
B N  2PCCB                 68    0.177  25.869   1.613   0.970   0.001   0.030
B M  2PCCB                 69    0.017   3.322   1.492   0.970   0.001   0.030
E S  2PCCB                 70    0.304  35.570   1.723   0.970   0.001   0.030
E E  2PCCB                 71    0.416  72.658   1.879   0.970   0.001   0.030
B Y  2PCCB                 72    0.058  12.352   1.093   0.923   0.002   0.076
B L  2PCCB                 73    0.048   8.862   0.219   0.858   0.002   0.139
E T  2PCCB                 74    0.474  65.688   1.222   0.694   0.003   0.303
E N  2PCCB                 75    0.333  48.678   0.704   0.176   0.004   0.820
B P  2PCCB                 76    0.103  14.559  -0.301   0.053   0.005   0.942
E K  2PCCB                 77    0.639 131.545   0.620   0.053   0.005   0.942
E K  2PCCB                 78    0.541 111.304  -0.066   0.058   0.017   0.925
E Y  2PCCB                 79    0.317  67.722  -0.449   0.053   0.043   0.903
E I  2PCCB                 80    0.329  60.939  -0.060   0.018   0.088   0.893
E P  2PCCB                 81    0.508  72.114  -1.478   0.018   0.047   0.935
E G  2PCCB                 82    0.585  46.039  -1.966   0.005   0.015   0.979
B T  2PCCB                 83    0.229  31.790  -2.222   0.018   0.088   0.893
E K  2PCCB                 84    0.370  76.191  -0.449   0.018   0.047   0.935
B M  2PCCB                 85    0.106  21.231  -0.292   0.018   0.047   0.935
E A  2PCCB                 86    0.316  34.867  -0.889   0.018   0.047   0.935
B F  2PCCB                 87    0.309  61.996  -1.659   0.018   0.019   0.964
E G  2PCCB                 88    0.460  36.194  -0.669   0.005   0.015   0.979
E G  2PCCB                 89    0.590  46.464  -1.229   0.005   0.015   0.979
B L  2PCCB                 90    0.104  19.061   0.018   0.005   0.015   0.979
E K  2PCCB                 91    0.499 102.665  -0.169   0.003   0.003   0.994
E K  2PCCB                 92    0.557 114.616   1.431   0.003   0.003   0.994
E E  2PCCB                 93    0.590 103.073  -0.013   0.858   0.002   0.139
E K  2PCCB                 94    0.619 127.246   1.128   0.923   0.002   0.076
B D  2PCCB                 95    0.178  25.650   0.740   0.923   0.002   0.076
B R  2PCCB                 96    0.072  16.419   1.135   0.970   0.001   0.030
E N  2PCCB                 97    0.393  57.579   0.930   0.970   0.001   0.030
B D  2PCCB                 98    0.232  33.374   1.696   0.970   0.001   0.030
B L  2PCCB                 99    0.019   3.516   1.605   0.970   0.001   0.030
B I  2PCCB                100    0.085  15.781   1.246   0.970   0.001   0.030
E T  2PCCB                101    0.295  40.944   1.435   0.988   0.000   0.012
B Y  2PCCB                102    0.137  29.362   1.406   0.970   0.001   0.030
B L  2PCCB                103    0.058  10.583   0.932   0.970   0.001   0.030
E K  2PCCB                104    0.388  79.729   1.612   0.923   0.002   0.076
E K  2PCCB                105    0.480  98.633   1.166   0.858   0.002   0.139
B A  2PCCB                106    0.194  21.423   0.326   0.600   0.003   0.397
E C  2PCCB                107    0.490  68.838  -0.201   0.246   0.004   0.750
E E  2PCCB                108    0.884 154.435   1.358   0.003   0.003   0.994

Explain the output.  Go back.
```

---
